# Facts label for transparent communication of AI Risks in mental health technology

**DOI:** 10.3389/fpsyt.2026.1887887

**Published:** 2026-08-13

**Authors:** Khatiya Moon, Matthew Tamura, J. R. Redmond, Gerry Craigen, Zuhal Haidari, Brooke Trainum, Darlene R. King

**Affiliations:** 1 Northwell, New Hyde Park, NY, United States; 2 Division of Engineering Science, Faculty of Applied Science & Engineering, University of Toronto, Toronto, ON, Canada; 3 Department of Sociology, New York University, New York, NY, United States; 4 Department of Psychiatry, Centre for Mental Health, University Health Network, Temerty Faculty of Medicine, University of Toronto, Toronto, ON, Canada; 5 American Psychiatric Association, Washington DC, United States; 6 Department of Psychiatry, University of Texas Southwestern Medical Center, Dallas, TX, United States; 7 Department of Clinical Informatics, Parkland Health, Dallas, TX, United States

**Keywords:** AI governance, artificial intelligence, health technology, mental health, risk communication, transparency

## Abstract

With interest in the adoption of artificial intelligence (AI)-enabled digital mental health technologies (AI-DMHTs) among the general population ceaselessly escalating, mental health clinicians are obliged to confront questions about their utility and safety for their practice. However, little guidance exists for developers on how to communicate risks to clinicians who may need to evaluate products for individuals with mental health concerns, individuals who are frequently vulnerable to such risks. We propose a standardized facts label for AI-DMHTs designed to enhance transparency and awareness about these tools and their risks to users, patients, and clinicians. This framework was developed by a multidisciplinary team from the American Psychiatric Association Committee on Mental Health Information Technology through iterative expert review and external clinician consultation, drawing upon existing scholarship in risk communication and informed consent, as well as international AI governance frameworks. The resulting facts label framework is composed of 8 sections: key identifying information, intended use, warnings, risks and limitations, model information, clinical evidence, accessibility and usability considerations, and privacy and security. This research represents a practical step toward responsible utilization of AI-DMHTs and aims to serve as a foundation for continued multidisciplinary collaboration regarding the development and governance of AI risk communication in the domain of mental health and healthcare more broadly.

## 1 Introduction

AI is rapidly reshaping consumer and clinical experiences of mental healthcare (1). Large Language Models (LLMs) are increasingly used for self-help, with 33% of teenagers using AI companions for social interaction and 12% specifically for emotional or mental health support (2, 3). Clinical AI is becoming common, promising operational support and streamlined workflows, with some AI-enabled technologies gaining Food and Drug Administration (FDA) authorization for clinical use in treatment and decision support (4, 5). While these advancements offer unprecedented opportunities for improving access and care (6), rapid integration introduces profound and often unaddressed risks.

Individuals seeking mental health support may be especially susceptible to potential AI harms, such as misinformation, algorithmic bias, or data insecurity (7). AI-driven misinformation can lead to dangerous outcomes, including inappropriate recommendations, suicide risk underestimation, and potentially psychosis (8–11). Privacy breaches of sensitive mental health data from LLMs, which may not be subject to HIPAA regulation, can have long-lasting repercussions (12). The potential for emotional manipulation, social isolation, and the induction of delusional states underscores the vulnerabilities specific to users with mental health concerns (13, 14). Within clinical settings, these complexities necessitate rigorous attention to informed consent, preservation of the therapeutic relationship, and management of crisis situations (15). Developers and regulators may overlook such considerations, opening patients up to serious risks (16).

A critical concern is the widespread lack of transparency surrounding AI-DMHTs. Insufficient and inaccessible user-oriented information leaves clinicians and patients unable to make informed decisions. A 2021 STAT investigation found that among 161 recently FDA-cleared AI-based medical devices, only 7 disclosed the racial breakdown of their training data, and only 13 disclosed gender demographics (17). A 2022 study found 24 out of 27 mental health app privacy policies required at least a college-level education to understand them (18). A study investigating privacy policies of AI-DMHTs revealed just 68% of apps have publicly accessible privacy policies, 25% are generic and cover no app-related scope, and only 11.2% mention the use of user health data for training AI systems (19). These gaps in disclosure underscore the need for improved AI risk communication.

Several international governance frameworks have begun to address this at a regulatory level. The U.S. FDA, Health Canada, and UK MHRA jointly identified transparency as a core principle of Good Machine Learning Practice (GMLP), calling for AI medical devices to provide clear, contextually relevant information on intended purpose and clinical integration (20). The EU AI Act mandates transparency disclosures for high-risk AI systems (21). The NIST AI Risk Management Framework (AI RMF) recommends multidisciplinary oversight and iterative risk assessment throughout the AI lifecycle (22). While these frameworks mandate transparency, they lack a practical, standardized mechanism for communicating information directly to clinicians and patients, particularly in regarding mental health.

Successes in addressing these gaps for AI generally have demonstrated that standardized transparency frameworks can achieve widespread adoption. One of the earliest efforts in AI transparency, the Dataset Nutrition Label, established standardized disclosure of information regarding datasets used in training (23). Model cards have become a *de facto* standard for communicating AI model characteristics, use considerations, and limitations (24). The George Washington Law Review identified an analogous approach of using a “facts label” framework for medical devices, where listing core information pertaining to the AI would reduce risks of bias and increase autonomous decisions (25). The healthcare community has begun developing health-AI facts labels, including the clinician-targeted Duke Model Facts Label (26), the more technical CHAI Nutrition Label (27), and Bramstedt’s patient-centered AI facts label (28). Empirical studies have found support for facts label approaches, finding that a holistic documentation approach supports patients in trusting and making informed AI-related healthcare decisions (29), and providing information on provider oversight, regulatory approval and performance can increase user trust and intention to use the device (30).

However, these frameworks contain key limitations for mental healthcare. The Duke and CHAI frameworks contain complex metrics and jargon, proving difficult for typical clinicians and patients (31). Current frameworks stop short of broader technological and relational contexts, with Bramstedt’s label lacking guidance on populating individual fields, hindering implementation, while others lack practical considerations of clinical workflows, third-party data handling, and safeguards for vulnerable populations. The need for a concise and effective framework to communicate the purposes, risks, warnings, and contextual considerations of AI-DMHTs to clinicians and patients thus remains.

To address this gap, we propose a novel framework. Building upon efforts within the American Psychiatric Association (APA), including the App Advisor framework for evaluating mental health applications (32), this paper proposes a *facts label framework* for AI-DMHTs. Iteratively developed by a multidisciplinary APA working group, this framework aims to enhance transparency, standardize risk disclosure, and provide actionable, context-specific information for patients and clinicians. Through novelty, specificity, and breadth, this theoretically informed framework offers a step towards ensuring patient safety and clinical integrity in the landscape of AI-DMHTs, with empirical validation identified as a next critical step.

## 2 Motivation

### 2.1 Facts label development

As members of the American Psychiatric Association’s (APA) Committee on Mental Health Information Technology (MHIT), we developed this facts label framework to provide a clear, standardized means of communicating information on limitations, risks, and considerations for use of AI-DMHTs.

This facts label was developed by a multidisciplinary North American team of seven members — three with backgrounds in psychiatry, two in law, one in public health, one in engineering, and one in sociology — and was initiated following an open call by the MHIT committee for the creation of an AI-DMHT facts label akin to broader medical AI guidelines (25). Given the diverse considerations needed to ensure comprehensiveness and sensitivity, this framework was only able to be constructed by bridging gaps between member’s disciplinary expertise in risk communication scholarship, regulatory guidance, and mental health and AI ethical concerns. Refinement was iterative and cumulative: a 2025 American Psychiatric Association annual meeting presentation and Q&A precipitated an expansion of the information provided to the user. An exploratory survey at an APA webinar confirmed relevance through questions such as, “if your patient wanted to find out more information about AI-DMHTs, would you know where to direct them to?”, while a presentation and Q&A for the APA Ethics Committee and an MHIT committee review yielded further direct expert clinical input. Although these processes informed and refined the framework, formal empirical validation remains a priority for future work.

The framework’s creation and development is notably grounded in emerging scholarship and global AI governance efforts, drawing upon growing calls for transparency to end-users and multidisciplinary oversight in AI development, as outlined in the GMLP for Medical Device Development: Guiding Principles; the NIST AI RMF; and the EU AI Act (20–22). The structure of information within the facts label was shaped by academic literature on risk communication (33–35), information sharing practices in informed consent and health literacy (36–40), and existing facts label scholarship (26–31).

### 2.2 Principles & priorities of design

Beyond existing work, the facts label is grounded in core principles tailored to the nature of AI in mental healthcare. We drew on several comparative analyses identifying principles across AI frameworks (41, 42), then added context with mental healthcare ethical directives such as informed consent, safeguards for vulnerable populations, and integrity of the therapeutic relationship (15, 43). Five core principles, operationalized in the facts label, emerged. *1. Equity and non-discrimination:* AI systems can perpetuate and amplify health disparities, operationalized by naming populations and clinical presentations missing from AI development, target populations and clinical use cases, and accessibility and usability. *2. Privacy and security:* AI-DMHTs must protect sensitive patient data, operationalized with information on privacy and security performance. *3. Accountability and professional responsibility:* healthcare provider and developer decisions must be grounded in clinical evidence, awareness of risks, and accountability, operationalized through fields detailing available clinical evidence and considerations for dangerous situations. *4. Transparency and explainability:* informed decisions require appropriate communication of risks and outcomes, operationalized by detailing different considerations for clinicians versus patients alongside core model information, warnings, and risks. *5. Human oversight and control:* AI must be controlled to mitigate harm, operationalized through fields detailing HIPAA compliance and user and clinician information control.

## 3 Facts label

The high-level structure of our facts label is shown in **Table 1**. Section 3.2 defines and describes the content of each information field. Recognizing distinct information needs and varying health and technical literacy among users and clinicians, our single facts label provides a layered approach with essential, comprehendible information at its primary level, designed for a 6th-grade equivalent reading level (37, 39, 40), and more clinically and technically detailed information separately accessible, ensuring both user approachability and clinician depth. These additional details are italicized and asterisked in **Table 1**. Tiered access allows individuals to engage with information relevant to their needs, with more granular details available for those, especially clinicians, who may require a deeper understanding of the system (30).

**Table 1:** A high-level view of the facts label.

Facts label section	Section content
Key Identifying Information	Developer Information. Clinician/User Development Involvement. Version/Date. Contact Information. Medical Device Status. Cost/Insurance Coverage.
Intended Use	Technology Description. Technology Explanation. Medical Supervision. Serious Mental Health Appropriateness. Target Population. Intended Context.
Warnings	Appropriateness in Crisis. Dangerous Situations. Fallback Resources.
Risks & Limitations	Performance. Disparities Across Population. Risks of Harm. Dataset Limitations.
Model Information	Model Development. Model Architecture/Design*. Model Interpretability*.
Clinical Evidence	Research Basis. Clinical Evaluation. Clinical Studies*.
Accessibility & Usability	Cultural Considerations. Visual Accessibility. Cognitive Accessibility. Digital Literacy. Internet Requirements. Usability Testing*.
Privacy & Security	Data Collection and Use. Data Storage and Security. Data De-Identification and Anonymization. 3rd Party Data Sharing. User Rights and Controls. Unforeseen Consequences and Mitigation. Handling of Protected Health Information (PHI)*. HIPAA Compliance*. Clinician Control*.

The contents of the facts label are outlined below. Italicised and asterisked contents indicate additional information most useful for clinicians outlined above.

The following section describes the eight sections of the facts label, providing the rationale for each section and details intended to populate each field.

### 3.1 Key identifying information

This section provides context about the technology and its development. Transparency and accountability begins with basic details such as support contact information, then extends to practical features such as medical device status and insurance coverage to help users make informed decisions about the suitability of the technology for their circumstances.

Developer Information: Who developed this technology?

Clinician/User Development Involvement: Were clinicians and individuals with lived experience involved in the development?

Version/Date: When was the technology released or last updated?

Contact Information: Who can be contacted with concerns, feedback, or questions?

Medical Device Status: Is this a medical device?

Costs/Insurance Coverage: Are there costs associated with use, and can these be covered by insurance?

### 3.2 Intended use

This section guides clinicians and users in determining whether the technology is appropriate for a given clinical context. The technological description and explanation provides a baseline understanding of what the model does, while the remaining fields narrow down appropriateness for specific vulnerable groups.

Technology Description: How would one describe the technology in a brief plain-language description?

Technology Explanation: What does the technology do? At a high-level, how does it work?

Medical Supervision: Is this technology meant to be used under the supervision of a healthcare professional?

Serious Mental Health Appropriateness: Is this technology appropriate for individuals with a serious mental illness?

Target Population: Is this technology meant to support any specific populations? Is it appropriate for children?

Clinical Population: Is this technology meant for a clinical population, a subclinical population, or both?

Intended Context: How is the technology meant to be used, and in what context? Is there a specific clinical context for the use of this technology?

### 3.3 Warnings

This section flags acute safety concerns, particularly for mental health. This section ensures clinicians and users are informed of how technology should not be used and what resources should be consulted if the user finds themselves outside of parameters of safe use.

Appropriateness in Crisis: Is the technology appropriate to use during a crisis?

Dangerous Situations: Could the AI system generate outputs or responses that could place a user in a dangerous situation?

Fallback Resources: What resources and information should be provided if a crisis arises that is outside the technology’s scope?

### 3.4 Risks and limitations

This section elaborates on appropriateness by identifying risks, drawing on model development information to ensure accountability.

Performance: What is the model’s performance across studies?

Disparities Across Population: Was the technology trained on data that reflects diverse backgrounds and identities, and if so which populations? Could some groups of users be harmed by responses that do not reflect their realities?

Risk of Harm: Is there any evidence or studies of harms associated with use of this type of technology? Could the technology cause or result in behavior including neglect of other human social supports or responsibilities, or even psychosis or death?

Dataset Limitations: Are there key demographic (race, ethnicity, gender, age, socioeconomic status, cultural background, etc.) or clinical presentations that are missing from the model’s development?

### 3.5 Model information

This section details the design of the AI model, helping clinicians evaluate rigor and clinical appropriateness.

Model Development: Was there any medical expertise insight in the development of the model?

Model Architecture/Design: What type of model is it? What dataset(s) were used to develop the model? What are the input and output features of the model?

Model Interpretability: Does the model employ interpretability methods? If so, what are the results and limitations of these methods?

### 3.6 Clinical evidence

This section identifies availability and quality of evidence of efficacy, helping clinicians and users understand the limits of any claims regarding benefits and risks.

Research Basis: Describe the research basis for the technology, if any. If none exists, this should be explicitly stated. What are the patient populations the product was studied for?

Clinical Evaluation: Has there been any clinical testing on this model? If so, what are the summarized results?

Clinical Studies: If clinical testing was done, what were the methodologies and performance metrics?

### 3.7 Accessibility & usability

This section helps clinicians and users determine whether the technology is accessible and appropriate for different circumstances.

Cultural Considerations: Has this technology been verified, tested, or designed for cross-cultural applications?

Visual Accessibility: Does the technology have any accessibility mechanisms for visual impairments (e.g., text-to-voice, changes in font size)?

Cognitive Accessibility: Does the technology have an accessibility mechanism for cognitive impairments (e.g., use of clear and simple language)?

Digital Literacy: How usable is the technology for individuals with low digital literacy?

Internet Requirements: Does the technology require internet access?

Usability Testing: Was usability testing done? How is usability feedback provided and handled?

### 3.8 Privacy & security

This section informs clinicians and users about how data is collected, stored, and handled.

Data Collection & Use: What information does the technology collect, and why? How is this data used (e.g., to personalize content, to improve the technology, for research)? Is there an accessible, accurate, and readable privacy policy explaining these practices in detail?

Data Storage & Security: Where is user data stored? What security measures are in place to protect data from unauthorized access or breaches?

Data De-Identification & Anonymization: Does the app de-identify or anonymize user data? If so, how?

Third-Party Data Sharing: Is data shared with any third parties (e.g., advertisers, researchers, service providers)? If so, with whom, for what purposes, and with what level of user consent? What control do users have over this data sharing?

User Rights & Controls: Can users opt out of data collection? Can users access, correct, or delete their data? How is data retention handled (e.g., how long is data kept, under what circumstances is it deleted)?

Unforeseen Consequences & Mitigation: What steps are taken to mitigate potential risks like social profiling, discrimination, or impact on insurability? How are user concerns and complaints addressed?

Handling of Protected Health Information (PHI): How is PHI stored, handled, and protected?

HIPAA Compliance: Is PHI handled in a manner that is compliant with HIPAA?

Clinician Control: Does the clinician have any control over how the data is shared? Can they see and override AI recommendations?

## 4 Example facts label

**Figure 1:**
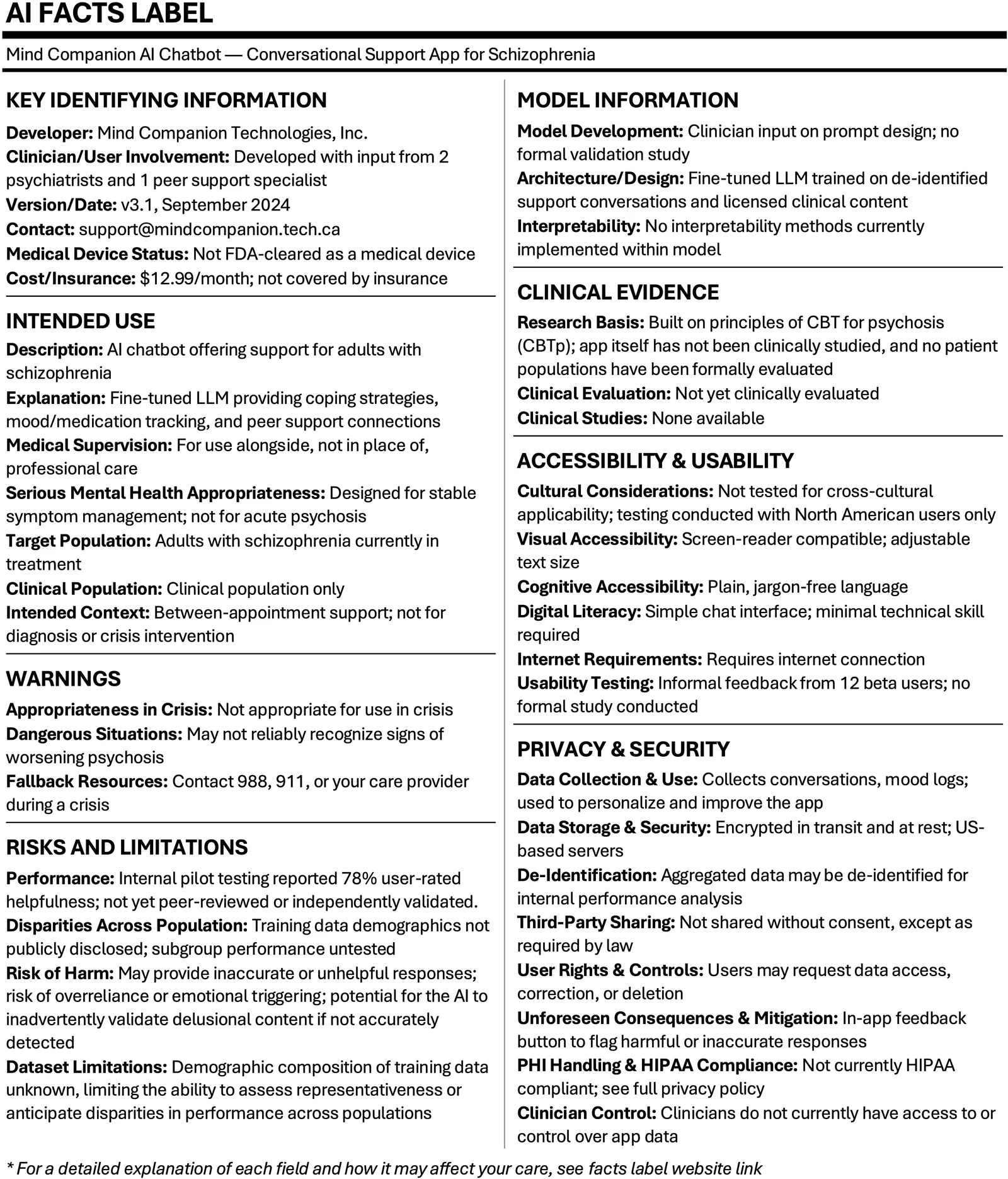
Example facts label for a hypothetical conversational AI-enabled support app for users with schizophrenia.

To demonstrate what the facts label looks like, a filled facts label for a hypothetical conversational AI app for support of users with schizophrenia is depicted in **Figure 1**.

## 5 Discussion

Aligning with regulatory guidance, core ethical principles, and existing scholarship, we propose a standardized AI facts label for digital mental health users and clinicians designed to enhance transparency and awareness, something international governance frameworks have established a clear, yet until now unfulfilled mandate for.

Some limitations require acknowledgement. The willingness of developers to provide potentially unfavorable label information may vary, and adoption would presently remain at developer discretion. We envision that a facts label will encourage accountability by making explicit what information should be available, and developers who demonstrate transparency will benefit from increased trust and adoption. Considering recent successes regulating AI, governments, once given a framework, will also be better equipped to require AI-DMHT developers to maintain transparency.

Second, feasibility populating certain fields may vary, as developers may lack the knowledge needed to measure and report AI performance and risks. However, existing regulatory frameworks already recommend or necessitate structured evaluation mechanisms that would generate much of the needed information. The NIST AI RMF’s MEASURE function recommends ongoing testing, evaluation, verification, and validation (22), while the EU AI Act mandates conformity assessment and re-assessment for high-risk AI systems (21). The facts label may not necessarily impose new burdens on developers, but rather provide a structured means of communicating information. The label is further designed as a living document, intended to be updated as models evolve, consistent with existing governance.

Ongoing challenges require further exploration to ensure the facts label functions meaningfully, rather than as a superficial compliance. Difficulties include independent verification of developer information, effective label updates for rapidly evolving AI systems, and communication regarding distinct regulatory burdens or cross-jurisdictional differences for developers. Consulting stakeholders such as developers who would be involved in facts label creation will be critical.

Future work should empirically evaluate usability and effectiveness in improving understanding and decision-making through usability testing, consistent with established co-design methodologies for digital mental health intervention (44–46). Our framework development is multidisciplinary and incorporates a wide variety of perspectives, yet core populations deserve consultation, including patients, non-clinical caregivers, advocacy groups, and developers whose perspectives could enrich the facts label design.

We issue a call to action for developers and regulators to critically assess and integrate this framework into operational and policy development processes. Prioritizing the safety, autonomy, and equitable care of mental health patients in this evolving technological era requires standardized, meaningful disclosure. This facts label offers a practical step towards ensuring AI mental health innovation is aligned with clinical responsibility and patient wellbeing.

## Data Availability

The original contributions presented in the study are included in the article/supplementary material. Further inquiries can be directed to the corresponding author.

## References

[1] Moon KC Sapra M Alvarado GL . Not the droids we’re looking for? Considering mental health apps from a disruptive innovation perspective. Ps. (2024) 75:602–3. doi: 10.1176/appi.ps.20230320 38823024

[2] Robb MB Mann S . Talk, Trust and Trade-Offs: How and Why Teens Use AI Companions. San Francisco, CA: Common Sense Media (2025). Available online at: https://www.commonsensemedia.org/sites/default/files/research/report/talk-trust-and-trade-offs_2025_web.pdf (Accessed April 15, 2026).

[3] Zao-Sanders M . How People are Really Using Gen AI in 2025 (2025). Available online at: https://hbr.org/2025/04/how-people-are-really-using-gen-ai-in-2025 (Accessed October 31, 2025).

[4] Auf H Svedberg P Nygren J Nair M Lundgren LE . The use of AI in mental health services to support decision-making: Scoping review. J Med Internet Res. (2025) 27:e63548. doi: 10.2196/63548 39854710 PMC11806275

[5] U.S. Food and Drug Administration . Artificial Intelligence-Enabled Medical Devices (2025). FDA. Available online at: https://www.fda.gov/medical-devices/software-medical-device-samd/artificial-intelligence-enabled-medical-devices (Accessed November 6, 2025).

[6] Limpanopparat S Gibson E Harris DA . User engagement, attitudes, and the effectiveness of chatbots as a mental health intervention: A systematic review. Comput Hum Behavior: Artif Humans. (2024) 2:100081. doi: 10.1016/j.chbah.2024.100081 38826717

[7] Timmons AC Duong JB Simo Fiallo N Lee T Vo HPQ Ahle MW . A call to action on assessing and mitigating bias in artificial intelligence applications for mental health. Perspect Psychol Sci. (2023) 18:1062–96. doi: 10.1177/17456916221134490 36490369 PMC10250563

[8] Levkovich I Elyoseph Z . Suicide risk assessments through the eyes of ChatGPT-3.5 versus ChatGPT-4: Vignette study. JMIR Ment Health. (2023) 10:e51232. doi: 10.2196/51232 37728984 PMC10551796

[9] McIntyre RS . Does the use of generative AI chatbots by patients introduce risk of adverse drug events? Expert Opin Drug Saf. (2025) 24:1249–51. doi: 10.1080/14740338.2025.2493351 40220270

[10] Østergaard SD . Will generative artificial intelligence chatbots generate delusions in individuals prone to psychosis? Schizophr Bull. (2023) 49:1418–9. doi: 10.1093/schbul/sbad128 37625027 PMC10686326

[11] Kolding S Lundin RM Hansen L Østergaard SD . Use of generative artificial intelligence (AI) in psychiatry and mental health care: A systematic review. Acta Neuropsychiatr. (2024) 37:e37. doi: 10.1017/neu.2024.50 39523628 PMC13130260

[12] Li J . Security implications of AI chatbots in health care. J Med Internet Res. (2023) 25:e47551. doi: 10.2196/47551 38015597 PMC10716748

[13] Phang J Lampe M Ahmad L Agarwal S Fang CM Liu AR . Investigating affective use and emotional well-being on ChatGPT. In: Arxiv. Ithaca, NY: Cornell University (arXiv) Preprint posted online April 4, 2025. doi: 10.48550/arXiv.2504.03888

[14] Østergaard SD . Emotion contagion through interaction with generative artificial intelligence chatbots may contribute to development and maintenance of mania. Acta Neuropsychiatr. (2025) 37:e79. doi: 10.1017/neu.2025.10035 40843483 PMC13130320

[15] Tavory T . Regulating AI in mental health: Ethics of care perspective. JMIR Ment Health. (2024) 11:e58493. doi: 10.2196/58493 39298759 PMC11450345

[16] Palmer A Schwan D . Digital mental health tools and AI therapy chatbots: A balanced approach to regulation. Hastings Cent Rep. (2025) 55:15–29. doi: 10.1002/hast.4979 40557918 PMC12817016

[17] Ross C . Explore STAT’s Database of FDA-Cleared AI Tools. STAT. Available online at: https://www.statnews.com/2021/02/03/fda-artificial-intelligence-clearance-products/ (Accessed October 31, 2025).

[18] Iwaya LH Babar MA Rashid A Wijayarathna C . On the privacy of mental health apps. Empir Software Eng. (2022) 28:2. doi: 10.1007/s10664-022-10236-0 36407814 PMC9643945

[19] Javed Y Bhojanam S . Evaluating privacy policies of AI-powered mHealth iOS applications. J Am Med Inform Assoc. (2025) 32:1581–8. doi: 10.1093/jamia/ocaf130 40737353 PMC12451934

[20] U.S. Food and Drug Administration, Health Canada, Medicines and Healthcare products Regulatory Agency . Good Machine Learning Practice for Medical Device Development: Guiding Principles (2021). FDA. Available online at: https://www.fda.gov/medical-devices/software-medical-device-samd/good-machine-learning-practice-medical-device-development-guiding-principles (Accessed October 31, 2025).

[21] Regulation (EU) 2024/1689 of the European Parliament and of the Council of 13 June 2024 Laying down Harmonised Rules on Artificial Intelligence and Amending Regulations (EC) No 300/2008, (EU) No 167/2013, (EU) No 168/2013, (EU) 2018/858, (EU) 2018/1139 and (EU) 2019/2144 and Directives 2014/90/EU, (EU) 2016/797 and (EU) 2020/1828 (Artificial Intelligence Act) (Text with EEA Relevance) (2024). Available online at: http://data.europa.eu/eli/reg/2024/1689/ojhttps://data.europa.eu/eli/reg/2024/1689/oj (Accessed October 31, 2025).

[22] U.S. Department of Commerce . Artificial Intelligence Risk Management Framework (AI RMF 1.0). Gaithersburg, MD: National Institute of Standards and Technology (U.S (2023). NIST AI 100-1. doi: 10.6028/NIST.AI.100-1

[23] Chmielinski KS Newman S Taylor M Joseph J Thomas K Yurkofsky J . The Dataset Nutrition Label (2nd Gen): Leveraging context to mitigate harms in artificial intelligence. In: Arxiv. Ithaca, NY: Cornell University (arXiv). Preprint posted online March 10, 2022. doi: 10.48550/arXiv.2201.03954

[24] Mitchell M Wu S Zaldivar A Barnes P Vasserman L Hutchinson B . Model cards for model reporting. In: Proceedings of the Conference on Fairness, Accountability, and Transparency. FAT* ’19. Association for Computing Machinery (2019). p. 220–9. doi: 10.1145/3287560.3287596

[25] Gerke S . Nutrition facts labels” for artificial intelligence/machine learning-based medical devices—the urgent need for labeling standards Social Science Research Network. Available online at: https://papers.ssrn.com/abstract=4404252 (Accessed October 31, 2025). Preprint posted online February 1, 2023.

[26] Sendak MP Gao M Brajer N Balu S . Presenting machine learning model information to clinical end users with model facts labels. NPJ Digit Med. (2020) 3:41. doi: 10.1038/s41746-020-0253-3 32219182 PMC7090057

[27] Coalition for Health AI (CHAI) . CHAI Advances Assurance Lab Certification and ‘Nutrition Label’ for Health AI (2024). CHAI. Available online at: https://www.chai.org/blog/chai-advances-assurance-lab-certification-and-nutrition-label-for-health-ai (Accessed October 31, 2025).

[28] Bramstedt KA . Artificial intelligence (AI) facts labels: An innovative disclosure tool promoting patient-centric transparency in healthcare AI systems. J Med Syst. (2025) 49:78. doi: 10.1007/s10916-025-02216-w 40495026

[29] Yoo DW Stroud AM Zhu X Miller JE Barry B . Toward patient-centered AI fact labels: Leveraging extrinsic trust cues. In: DIS (Des Interact Syst Conf), New York, NY: Association for Computing Machinery (ACM). vol. 2025. (2025). p. 676–90. doi: 10.1145/3715336.3735758 PMC12461688 41019587

[30] Zhu X Stroud AM Minteer SA Yoo DW Ridgeway JL Mooghali M . Key information influencing patient decision-making about AI in health care: Survey experiment study. J Med Internet Res. (2026) 28:e75615. doi: 10.2196/75615 41525463 PMC12795307

[31] Gilbert S Adler R Holoyad T Weicken E . Could transparent model cards with layered accessible information drive trust and safety in health AI? NPJ Digit Med. (2025) 8:124. doi: 10.1038/s41746-025-01482-9 40000736 PMC11861263

[32] Lagan S Aquino P Emerson MR Fortuna K Walker R Torous J . Actionable health app evaluation: Translating expert frameworks into objective metrics. NPJ Digit Med. (2020) 3:100. doi: 10.1038/s41746-020-00312-4 32821855 PMC7393366

[33] Spiegelhalter D . Risk and uncertainty communication. Annu Rev Stat Appl. (2017) 4:31–60. doi: 10.1146/annurev-statistics-010814-020148 41139587

[34] Chmielinski K Newman S Kranzinger CN . The CLeAR Documentation Framework for AI Transparency: Recommendations for practitioners & context for policymakers (2024). Harvard Kennedy School, Shorenstein Center. Available online at: https://shorensteincenter.org/resource/clear-documentation-framework-ai-transparency-recommendations-practitioners-context-policymakers/ (Accessed October 31, 2025).

[35] Presenting Quantitative Information About Decision Outcomes: A Risk Communication Primer for Patient Decision Aid Developers - PMC. Available online at: https://pmc.ncbi.nlm.nih.gov/articles/PMC4045391/ (Accessed June 24, 2026). 10.1186/1472-6947-13-S2-S7 PMC4045391 24625237

[36] Bester J Cole CM Kodish E . The limits of informed consent for an overwhelmed patient: Clinicians’ role in protecting patients and preventing overwhelm. AMA J Ethics. (2016) 18:869–86. doi: 10.1001/journalofethics.2016.18.9.peer2-1609 27669132

[37] Martinez-Martin N Kreitmair K . Ethical issues for direct-to-consumer digital psychotherapy apps: Addressing accountability, data protection, and consent. JMIR Ment Health. (2018) 5:e9423. doi: 10.2196/mental.9423 29685865 PMC5938696

[38] Chau M Rahman MG Debnath T . From black box to clarity: Strategies for effective AI informed consent in healthcare. Artif Intell Med. (2025) 167:103169. doi: 10.1016/j.artmed.2025.103169 40446592

[39] Tucker CA . Promoting personal health literacy through readability, understandability, and actionability of online patient education materials. J Am Heart Assoc. (2024) 13:e033916. doi: 10.1161/JAHA.124.033916 38567677 PMC11262515

[40] Evaluating Adult Digital Health Literacy, 2020–2025: A Systematic Review | PLOS Digital Health. Available online at: https://journals.plos.org/digitalhealth/article?id=10.1371/journal.pdig.0001075 (Accessed June 24, 2026). 10.1371/journal.pdig.0001075 PMC12643288 41284673

[41] Fjeld J Achten N Hilligoss H Nagy A Srikumar M . Principled artificial intelligence: Mapping consensus in ethical and rights-based approaches to principles for AI. In: Social Science Research Network. Cambridge, MA, USA: Berkman Klein Center for Internet & Society (Harvard University). Preprint posted online January 15, 2020. doi: 10.2139/ssrn.3518482

[42] Corrêa NK Galvão C Santos JW Del Pino C Pinto EP Barbosa C . Worldwide AI ethics: A review of 200 guidelines and recommendations for AI governance. Patterns. (2023) 4:100857. doi: 10.1016/j.patter.2023.100857 37876898 PMC10591196

[43] Varkey B . Principles of clinical ethics and their application to practice. Med Princ Pract. (2021) 30:17–28. doi: 10.1159/000509119 32498071 PMC7923912

[44] Hawke LD Sheikhan NY Bastidas-Bilbao H Rodak T . Experience-based co-design of mental health services and interventions: A scoping review. SSM - Ment Health. (2024) 5:100309. doi: 10.1016/j.ssmmh.2024.100309 38826717

[45] Chinsen A Berg A Nielsen S Trewella K Cronin TJ Pace CC . Co-design methodologies to develop mental health interventions with young people: A systematic review. Lancet Child Adolesc Health. (2025) 9:413–25. doi: 10.1016/S2352-4642(25)00063-X 40311649

[46] Francis A Le A Adams-Leask K Procter N . Utilising co-design to develop a lived experience informed personal safety tool within a mental health community rehabilitation setting. Aust Occup Ther J. (2024) 71:1076–88. doi: 10.1111/1440-1630.12988 39129440 PMC11609345

